# Data on structural analysis of cholesterol binding and sterol selectivity by ABCG5/G8

**DOI:** 10.1016/j.dib.2022.108754

**Published:** 2022-11-17

**Authors:** Danny Farhat, Fatemeh Rezaei, Jyh-Yeuan Lee

**Affiliations:** Department of Biochemistry, Microbiology and Immunology, Faculty of Medicine, University of Ottawa, Ottawa, Ontario, Canada

**Keywords:** ABCG5, ABCG8, Cholesterol, Sitosterol, Stigmasterol, X-ray crystallography, Molecular docking

## Abstract

ATP-Binding cassette subfamily G (ABCG) sterol transporters maintain whole body endogenous and exogenous sterol homeostasis. A substantial portion of exogenous sterols are undigestible phytosterols (plant sterols), which can introduce complications when accumulated. ABCG5/G8 is the main protein functioning to remove ingested plant sterols providing protection from their toxic effects, although, the structural features behind substrate binding in ABCG5/G8 remain poorly resolved. Within this data article, we present extended preceding in the determination of the cholesterol-bound crystal structure and the sterol docking analysis. The crystal structure was deposited in the Protein Data Bank with the accession number of 8CUB, whereas the diffraction images were deposited at the SBGrid Data Bank. This dataset follows the research article entitled as “Structural analysis of cholesterol binding and sterol selectivity by ABCG5/G8” (doi: 10.1016/j.jmb.2022.167795).

**Specifications Table**

**Table:** 

Subject	Biological sciences
Specific subject area	Structural biology
Type of data	Figure, Table, External Depository
How the data were acquired	Figs. 2/3 & Tables 1/2: the data was acquired via X-ray crystallography by collecting the X-ray diffraction images at the beamline 19-ID at the Advanced Photon Source (APS). Fig. 4 & Table 3, Table 4, Table 5: the data was acquired via molecular docking using programs of UCSF Dock6.9 through the SBGrid Consortium. Fig. 5: the data was acquired via multiple sequence alignment using PSI-BLAST and PROMALS3D. Only five representative species were used in the figure.
Data format	Raw Analyzed
Description of data collection	The X-ray diffraction data were collected remotely at beamline 19-ID at the Advanced Photon Source. Diffraction images of a total of 3 crystals were merged for data processing. Data of the molecular docking were obtained by excluding published ligands in the input models. Amino acid sequences of top 300 mammalian species were obtained by the online-based PSI-BLAST. All sequences in FASTA format were subjected to multiple sequence alignment for conservation analysis.
Data source location	Protein Biophysics Core Facility, Faculty of Medicine, University of Ottawa, Ontario, Canada Advanced Photon Source (APS), Argonne National Laboratory, Lemont, Illinois, USA National Center for Biotechnology Information, National Institute of Health, USA
Data accessibility	1. Model of the crystal structure Repository name: Protein Data Bank (PDB) Data identification number: 8CUB Direct URL to data: https://doi.org/10.2210/pdb8CUB/pdb. 2. Raw X-ray images Repository name: SBGrid Data Bank Data identification number: 971, 972, 973 Direct URL to data: https://doi.org/10.15785/SBGRID/971, https://doi.org/10.15785/SBGRID/972, https://doi.org/10.15785/SBGRID/973).
Related research article	Structural analysis of cholesterol binding and sterol selectivity by ABCG5/G8 D. Farhat, F. Rezaei, M. Ristovski, Y. Yang, A. Stancescu, L. Dzimkova, S. Samnani, J.-F. Couture, J.-Y. Lee, Structural Analysis of Cholesterol Binding and Sterol Selectivity by ABCG5/G8., J. Mol. Biol. 434 (2022) 167795. https://doi.org/10.1016/j.jmb.2022.167795.

## Value of the Data

• The crystallographic data shows the electron density of a novel cholesterol ligand in the ABCG5/G8 crystal structure and the structural information at highest possible resolutions.
• The ligand docking data shows a collection of predicted poses of cholesterol, sitosterol and stigmasterol, the top 3 enriched sterols in Sitosterolemia patients. Contracting data via comparison of sterol poses between ABCG1 and ABCG5/G8 provides hypothesis-driven models for sterol selectivity by ABCG sterol transporters.
• The sequence analysis data shows a collection of amino acid conservation patterns on the transmembrane helices at the putative substrate translocation pathways in mammalian and yeast ABCG transporters. This provides the preliminary results to suggest a novel and key region within ABCG proteins that may play a role in regulating transporter activities and to guide structural and functional studies using experimental approaches.

## 1 Data Description

**Fig. 1:**
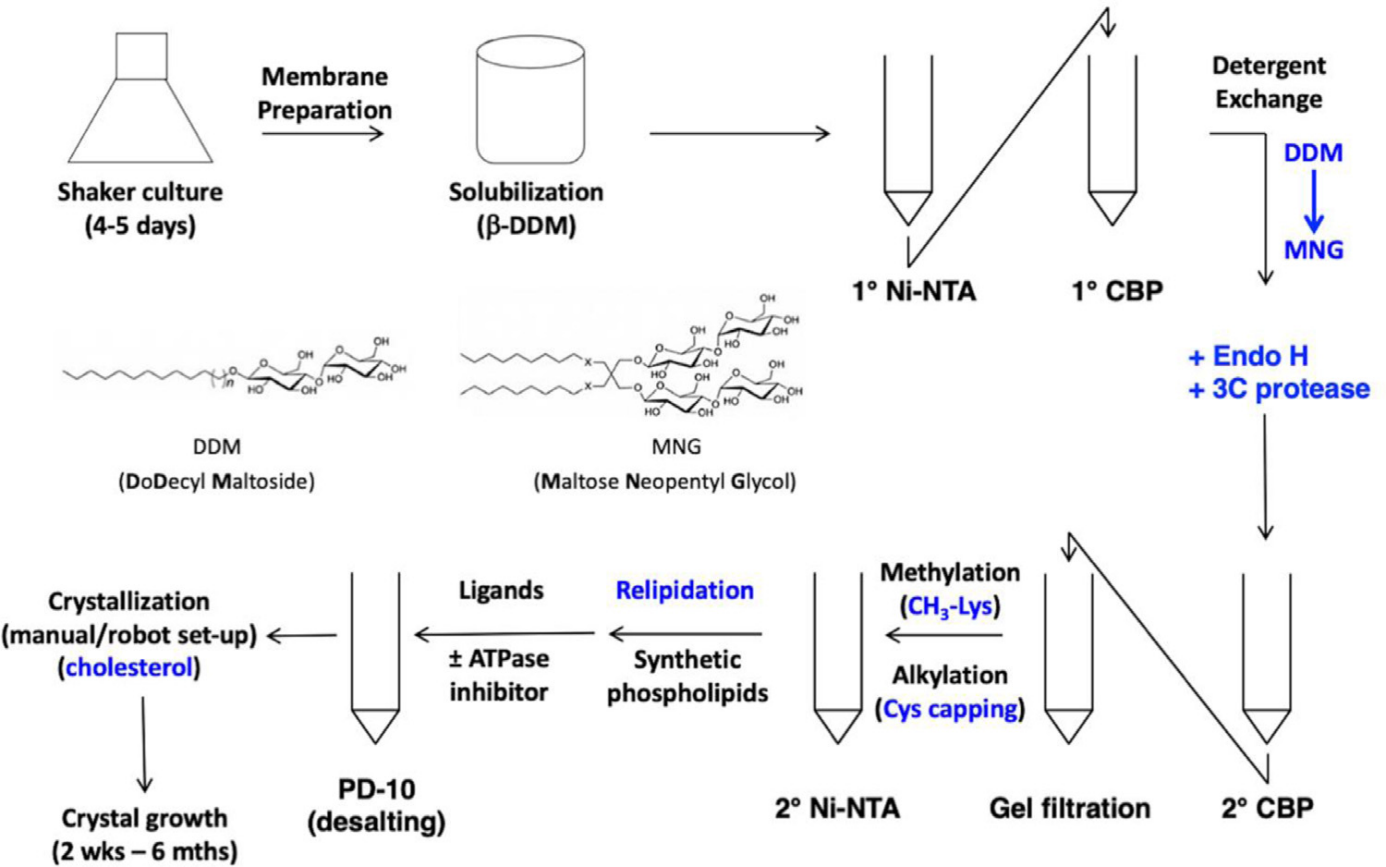
Schematic flowchart of ABCG5/G8 purification for bicelle crystallization. *(Top left → top-right → bottom-right → bottom-left)* The β-DDM-solubilized membrane preparation was subjected to a Ni-NTA/CBP tandem affinity column chromatography to extract ABCG5/G8 heterodimers and exchange detergents with MNG. Endo H and 3C protease were used to remove the extracellular N-glycans and the CBP tag, respectively. CBP-free proteins were collected via the flow-through of a 2^nd^ CBP column, which was further purified by gel-filtration chromatography. Purified transporters were subjected to lysine methylation and cysteine-capping alkylation, and the excess chemicals were filtered by a 2^nd^ Ni-NTA column. Synthetic phospholipids were added to the Ni-NTA bound proteins, and the eluates were then subjected to desalting by a PD-10 spin column. If needed, ligands, *e.g.*, ATPase inhibitors, were mixed with the 2^nd^ Ni-NTA eluates before desalting. The relipidated proteins were mixed with cholesterol in equilibrium and then concentrated to desired concentration for crystal growth.

**Fig. 2:**
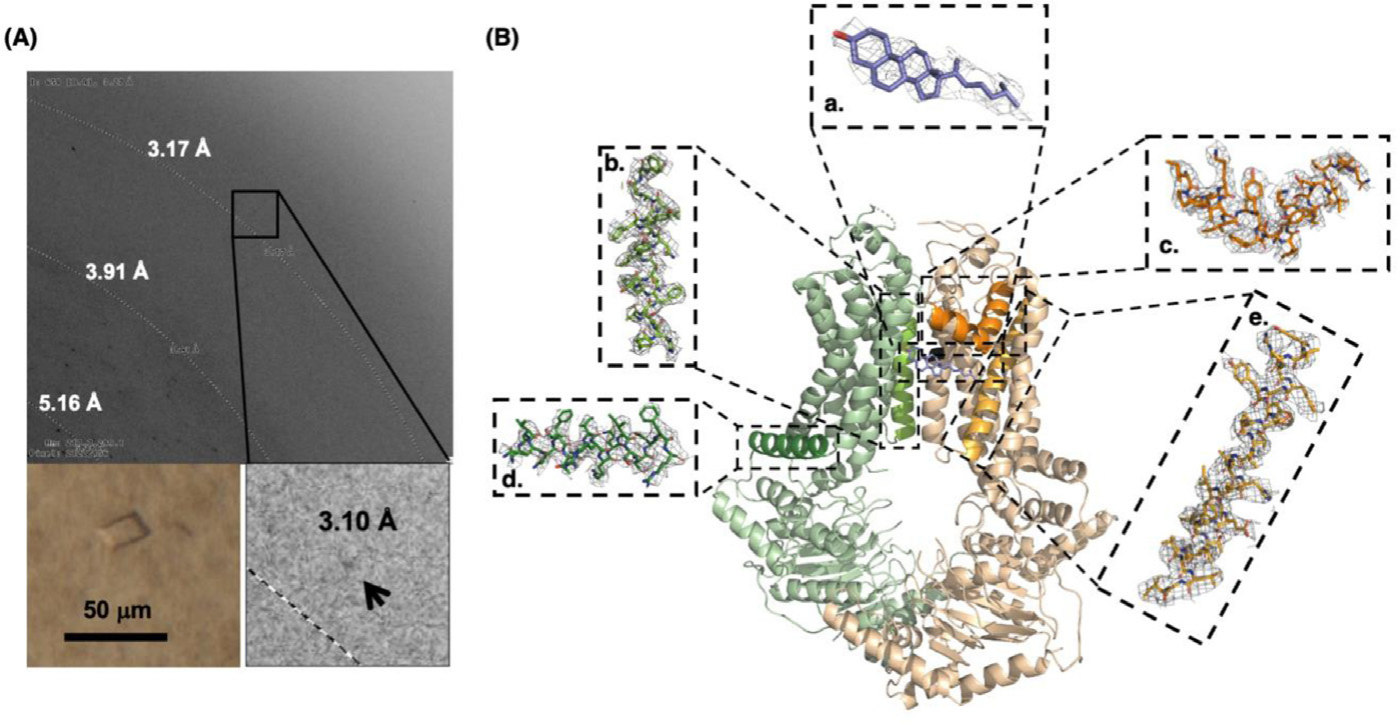
The x-ray diffractions, the crystal structure of cholesterol-bound ABCG5/G8, and the fitting of model to electron densities (2Fo-Fc omit map). **A.** An X-ray diffraction image of ABCG5/G8 bicelle crystals, showing up to 3.1 Å (bottom right). A typical bicelle crystal grew into a length of 25-50 micrometer (bottom left). **B.** The structure of ABCG5/G8 (green: ABCG8, tan: ABCG5) is shown in cartoon presentation (center). The cholesterol molecule (blue) was modeled to an orthogonal electron density at the upper part of the transporter-membrane interface and near the dimeric interface of the transmembrane domains. Black sphere indicates Alanine 540 (A540) residue of ABCG5, which contributes to the cholesterol-binding site. Selective segments of ABCG5/G8 proteins are illustrated by fitting the atomic model to the electron densities from the X-ray diffraction data, including cholesterol molecule **(a)**, ABCG8 transmembrane helix 5 (TMH5, light green) **(b)**, ABCG5 re-entry helix (orange) **(c)**, ABCG8 connecting helix (dark green) **(d)** and ABCG5 TMH4 (light orange) **(e).** The electron densities with a mesh presentation were contoured at 1 σ with a thickness of 1.5 as implemented in PyMOL).

**Fig. 3:**
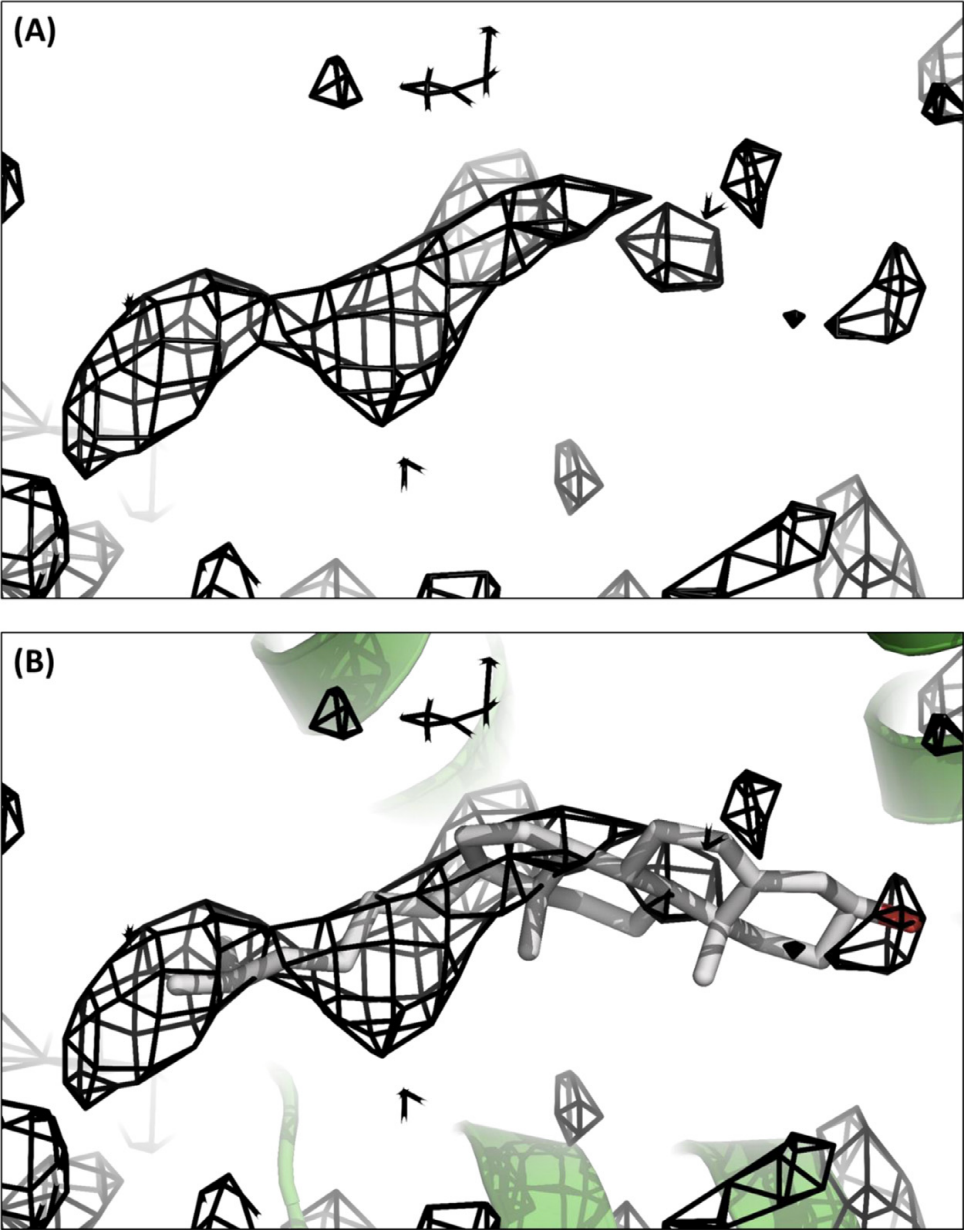
Fo-Fc omit map of a cholesterol molecule density. **A.** Fo-Fc omit map of a horizontal electron density observed in the structure of ABCG5/G8 (PDB ID: 8CUB). **B.** Stick image of a cholesterol molecule fitting in this density.

**Fig. 4:**
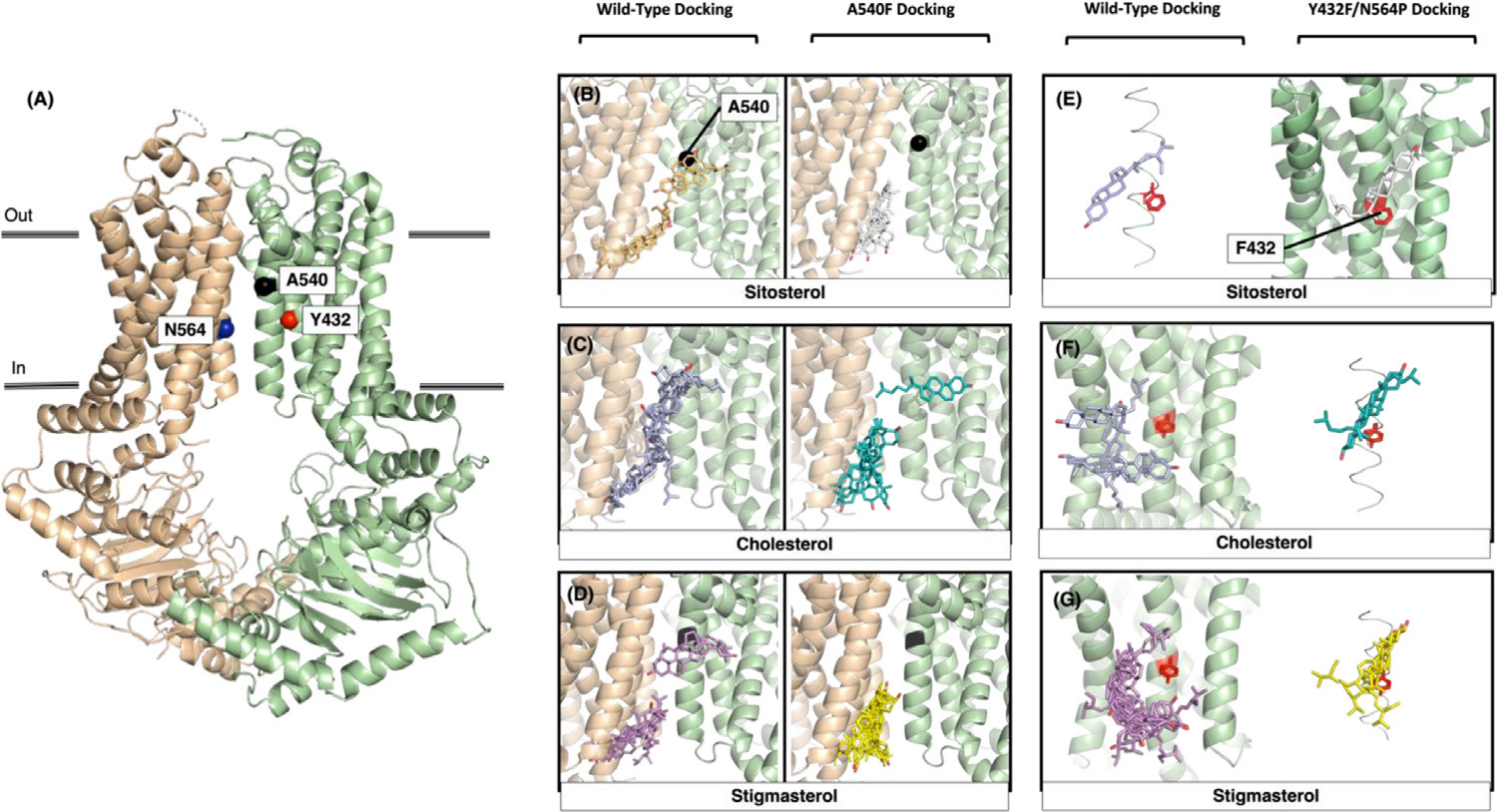
Docking of cholesterol and phytosterol (sitosterol and stigmasterol) on wild-type ABCG5/G8 as well as ABCG5_A540F_/G8 and ABCG5_Y432F_/G8_N564P_. **A.** crystal structure of ABCG5/G8 (green/tan, PDB ID: 8CUB) with mutation sites shown as spheres: Alanine 540 (black), tyrosine 432 (red), Asparagine 564 (blue). **B-D.***left. S*terol docking on ABCG5/G8 near residue A540. *Right.* Sterol docking on A540F mutant. **E-G.***left. S*terol docking on ABCG5/G8 near residue Y432. *Right.* Sterol docking on double mutant model.

**Fig. 5:**
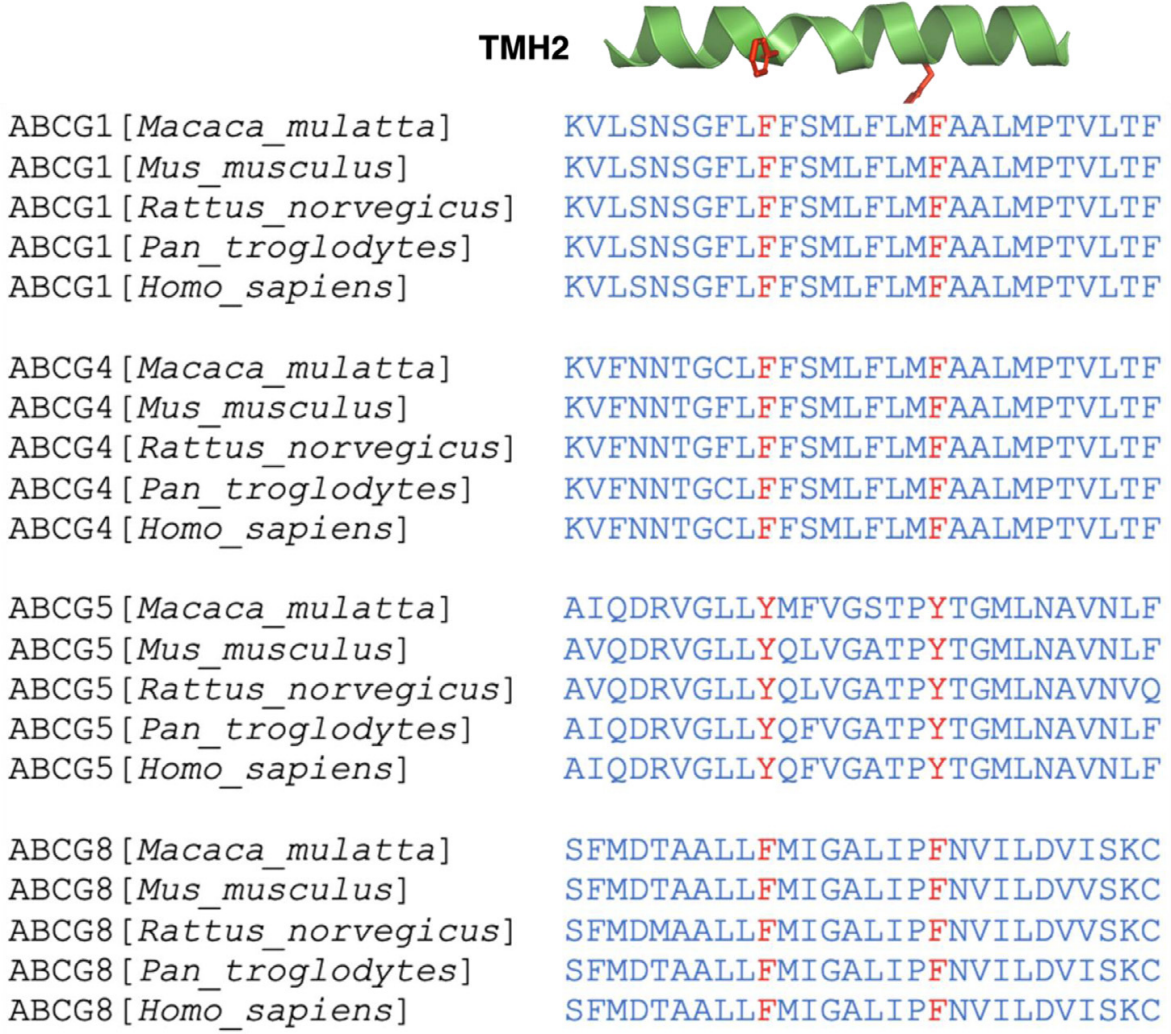
Sequence alignment of Phenylalanine Highway in transmembrane helix 2 (TMH2) of ABCG sterol transporters in different mammal species. Sequence analysis done through PROMALS3D. TMH2 within the transmembrane domain (TMD) of ABCG protein shown in blue while the phenylalanine's (and tyrosine in ABCG5) are shown in red. A sequence analysis was also performed on five different mammalian species: *Homo sapiens, Mus musculus, Macaca Mulatta, Rattus norvegicus* and *Pan troglodytes.* Transmembrane region of transmembrane helix 2 (TMH2) is shown in blue while the aromatic groups of the phenylalanine highway are shown in red.

The data reported herein describes the data acquisition, processing and presentation for the crystal structure of a cholesterol-bound conformation of ABCG5/G8 sterol transporter, including figures, tables, and external repositories. Fig. 1 depicts a flow-chart of protein purification leading to crystallization; Figs. 2 and 3 illustrate the atomic model of the crystal structure and electron density fittings to the protein backbone and the cholesterol ligand, where the sterol binding site is near alanine 540 of ABCG5/G8. Table 1 summarizes the X-ray reflection intensities of processed X-ray images, and Table 2 highlights the results of i) model searching by Phaser's molecular replacement and ii) model refinement by Phenix. At the **Protein Data Bank**, the refined and validated structural data is publicly available through the model accession number 8CUB. At the **SBGrid Data Bank**, three sets of the raw X-ray diffraction images are publicly accessible through the identification numbers of 971, 972, and 973. Fig. 4 shows the data on the molecular docking of cholesterol and two plant sterols (*i.e.*, sitosterol and stigmasterol) to ABCG5/G8 sterol transporters. Tables 3, 4 and 5 summarize the ligand-docking results on ABCG5/G8 by cholesterol, stigmasterol, and sitosterol, respectively. Fig. 5 highlights the amino acid conservation analysis of the Phenylalanine Highway (PH) motif on transmembrane helix 2 (TMH2) in five mammalian ABCG sterol transporters.

**Table 1:** Summary of reflections intensities and R-factors by shells.

	R linear = SUM (ABS(I - <I>)) / SUM (I)											
	R square = SUM ((I - <I>) ** 2) / SUM (I ** 2)											
	Chi**2 = SUM ((I - <I>) ** 2) / (Error ** 2 * N / (N-1)))											
	In all sums single measurements are excluded											
Shell	Lower	Upper	Average	Average		Norm.	Linear	Square				
limit	Angstrom		I	error	stat.	Chi**2	R-fac	R-fac	Rmeas	Rpim	CC1/2	CC*

	30.00	10.70	2274.5	122.9	30.2	1.132	0.112	0.146	0.118	0.035	0.975	0.994
	10.70	8.56	1586.6	85.0	27.1	0.924	0.109	0.125	0.114	0.032	0.995	0.999
	8.56	7.49	553.5	37.9	18.7	0.984	0.195	0.197	0.203	0.055	0.993	0.998
	7.49	6.82	248.7	23.3	15.4	1.008	0.412	0.410	0.428	0.113	0.977	0.994
	6.82	6.33	136.7	19.3	15.4	0.951	0.819	0.813	0.851	0.224	0.940	0.985
	6.33	5.96	95.8	18.6	16.0	0.938	1.339	1.335	1.390	0.363	0.882	0.968
	5.96	5.67	80.2	18.6	16.6	0.929	1.844	1.722	1.915	0.501	0.806	0.945
	5.67	5.42	72.9	19.8	18.1	1.025	2.159	2.243	2.240	0.582	0.748	0.925
	5.42	5.21	69.5	20.6	19.0	0.981	3.018	2.293	3.133	0.818	0.744	0.924
	5.21	5.04	76.6	21.8	20.0	0.991	2.555	2.151	2.653	0.695	0.730	0.919
	5.04	4.88	75.4	23.1	21.2	0.976	2.825	2.350	2.933	0.767	0.739	0.922
	4.88	4.74	72.1	23.8	22.1	0.983	3.770	2.698	3.914	1.025	0.693	0.905
	4.74	4.62	69.0	25.0	23.5	0.972	3.445	2.840	3.578	0.941	0.627	0.878
	4.62	4.50	63.7	25.7	24.2	0.993	4.890	3.254	5.078	1.333	0.624	0.877
	4.50	4.40	56.4	26.8	25.5	0.980	5.584	4.054	5.799	1.524	0.516	0.825
	4.40	4.31	51.1	27.3	26.2	0.994	6.411	4.457	6.658	1.748	0.428	0.774
	4.31	4.22	45.4	28.2	27.2	0.995	8.358	5.477	8.683	2.290	0.352	0.721
	4.22	4.14	43.1	28.3	27.5	0.966	7.890	5.972	8.194	2.156	0.305	0.684
	4.14	4.07	37.5	30.0	29.3	0.963	9.188	6.629	9.546	2.521	0.282	0.663
	4.07	4.00	30.7	30.9	30.5	0.972	0.000	8.120	0.000	2.958	0.156	0.520
All reflections			294.8	33.1	22.7	0.983	0.640	0.345	0.666	0.191	0.983	0.996
rmsdel,sdelf2,sumwxf			1.776540		440257.4		139494.3					
	0	0.9804	0.8806		-12.6607		1.7867		1.0977			
	1	0.9911	4.2551		0.0033		0.0130		0.0253			
	2	1.2273	0.1277

**Table 2:** Summary of Phaser solutions and Phenix refinement.

PHASER DATA					
PHASER MODULE			Molecular Replacement		

Solution-Group Name (Hall Symbol)			I 2 2 2		
Space Group Number			23		
Unit Cell (*a, b, c* (Å))			173.27 230.11 249.82 90.00 90.00 90.00		

MOLECULAR REPLACEMENT PACKING RESULTS					

Number of Solutions			2		
#in	#out	Clash-%	Space group	Annotation	

1	Top1	0	I 2 2 2	RFZ=5.9 TFZ=14.9 PAK=0	
2	2	0	I 2 2 2	RFZ=5.3 TFZ=13.0 PAK=0	

PHASER OUTPUT RESULTS					

#in	#out	Space Group	Start LLG Rval TFZ	Refined LLG Rval TFZ	

1	Top1	I 2 2 2	2744.9 49.7 31.5	2848.0 49.6 52.7	

PHENIX REFINEMENT INPUT SETTINGS					

Data labels		I(+), SIGI(+),I(-),SIGI(-)			
Refinement strategy		XYZ (reciprocal-space), XYZ (real-space), Individual B factor, TLS parameters, Occupancies, Automatically correct N/Q/H errors			
Scattering table		N_gussian			
Number of cycles		3			

FINAL REFINEMENT OUTPUT RESULTS					

R-work	R-free	Bonds	Angles	Ramachandran outliers	Isotropic B factor

0.2456	0.3027	0.005	0.919	0.56%	Min: 4:72, Max: 183.77, Mean: 54.06

**Table 3:** Cholesterol Docking on ABCG5/G8?. Docking data of bound cholesterol ligands to wild type (WT) and A540F mutant, ranked according to Van der Waal energy values in increasing order. Charges have units of electron charges while energies have units of kcal/mol. CLR: cholesterol.

Ligand-Model	Cluster size	Formal Charge	Grid Score	Electrostatic energy	Van der Waal Energy^	Rotatable Bonds
CLR WT	2	0.0031	-43.29	0.16	-43.45	6
	12	0.0031	-43.36	-0.25	-43.11	6
	2	0.0031	-42.86	0.008	-42.87	6
	2	0.0031	-42.56	0.08	-42.64	6
	1	0.0031	-42.52	-0.26	-42.26	6
	1	0.0031	-40.16	-0.23	-39.93	6
	3	0.0031	-40.10	-0.60	-39.49	6
	2	0.0031	-39.53	-0.37	-39.16	6

CLR A540F	4	-0.0005	-51.91	-0.38	-51.53	6
	1	-0.0005	-51.46	-0.07	-51.39	6
	12	-0.0005	-51.45	-0.36	-51.08	6
	4	-0.0005	-50.59	-0.54	-50.10	6
	1	-0.0005	-49.79	-0.34	-49.46	6
	3	-0.0005	-49.70	-0.79	-48.91	6

**Table 4:** Stigmasterol Docking on ABCG5/G8. Docking data of bound stigmasterol ligands to wild type (WT) and A540F mutant, ranked according to Van der Waal energy values in increasing order. Charges have units of electron charges while energies have units of kcal/mol. STG: stigmasterol.

Ligand-Model	Cluster size	Formal Charge	Grid Score	Electrostatic energy	Van der Waal Energy ^	Rotatable Bonds
STG WT	15	-0.0019	-49.38	0.04	-49.42	7
	1	-0.0019	-49.48	-0.70	-48.78	7
	3	-0.0019	-48.39	-0.17	-48.22	7
	4	-0.0019	-47.95	-0.83	-47.11	7
	1	-0.0019	-47.78	-0.67	-47.11	7
	1	-0.0019	-47.81	-3.48	-44.32	7

STG A540F	8	-0.0006	-58.43	-0.54	-57.89	7
	3	-0.0006	-55.66	-0.35	-55.31	7
	5	-0.0006	-54.71	-0.59	-54.11	7
	2	-0.0006	-53.90	-0.37	-53.51	7
	2	-0.0005	-53.10	-0.21	-52.89	7
	3	-0.0006	-52.90	-0.22	-52.67	7
	1	-0.0006	-52.43	-0.17	-52.27	7
	1	-0.0006	-52.41	-0.59	-51.82	7

**Table 5:** Sitosterol Docking on ABCG5/G8. Docking data of bound sitosterol ligands to wild type (WT) and A540F mutant ranked according to Van der Waal energy values in increasing order. Charges have units of electron charges while energies have units of kcal/mol.

Ligand-Model	Cluster size	Formal Charge	Grid Score	Electrostatic energy	Van der Waal Energy^	Rotatable Bonds
Sitosterol Wild-type	18	-0.0006	-50.78	-0.14	-50.64	7
	4	-0.0006	-49.42	-0.24	-49.18	7
	1	-0.0006	-48.45	-0.22	-48.22	7
	1	-0.0006	-47.67	0.26	-47.93	7
	1	-0.0006	-47.62	0.13	-47.75	7

Sitosterol A540F	20	-0.0006	-58.74	-0.17	-58.57	7
	3	-0.0006	-55.41	-0.29	-55.12	7
	1	-0.0006	-53.78	-0.21	-53.57	7
	1	-0.0006	-53.87	-0.46	-53.41	7

## 2 Experimental Design, Materials and Methods

### 2.1 Protein crystallization

Proteins used for crystallization were prepared according to the previously established protocol [4]. Fig. 1 highlights the key steps during the protein purification. The relipidated proteins were treated with 1 mM AMPPNP (sodium salt, Roche) for overnight at 4°C and desalted with a PD-10 column. The desalted and lipidated proteins were incubated overnight with cholesterol (prepared in isopropanol) to a final concentration of ∼20 µM. The protein precipitants were removed by ultracentrifugation, using a TLA-100 rotor and a benchtop Optima TLX ultracentrifuge at 100,000 g and 4°C for 10 minutes. The supernatants were concentrated to a final protein concentration of 30-50 mg/ml. The cholesterol-treated proteins were reconstituted into 10% DMPC/Cholesterol/CHAPSO bicelles. To prepare the 10% bicelle stock solution, lipids and detergents (CHAPSO) were mixed in a ratio of 3:1 (w/w), where the lipids contained 5 mol % cholesterol and 95 mol % DMPC. The proteins and bicelle stock were gently mixed in a 1:4 (v/v) ratio, resulting in a final protein concentration of ∼10 mg/ml. The protein/bicelle mixture was incubated on ice for 30 min and then mixed with equal-volume crystallization reservoir solution (usually 0.5 or 1mL). In a 20°C incubator, crystals grew by a hanging-drop vapor diffusion technique in the 48-well VDX48 crystallization trays using thin glass cover slides. The crystallization reservoir solution contained 1.8–2.0 M ammonium sulfate, 100 mM MES pH 6.5, 2–5% PEG400, and 1 mM TCEP. Within 1-2 weeks of incubation, the protein crystals (Fig. 2) were harvested by submerging with 0.2 M sodium malonate and flash-freezing in liquid nitrogen.

### 2.2 Collection of X-ray diffraction data

The X-ray diffraction images were collected on a Quantum 315r detector at beamlines 19-ID at the Advanced Photon Source (APS), at which the data collection was carried out remotely via SBCCollect GUI as the beamline control. The crystals were exposed to X-ray radiation (wavelength ∼ 1Å) under the cryogenic condition with the beam window fully open and without attenuation. For each crystal, 90 frames of X-ray diffraction images were collected at an incremental step of 1° and for 2-minute exposure (Fig. 2). Diffraction images from all crystals were individually processed by Denzo and XdisplayF as implemented in HKL2000. Each dataset were used to proceed to scaling by HKL2000’s Scalepack. Datasets of three crystals were merged for data processing, as they contained similar unit cell dimension, and were scaled to 4-30Å to ensure sufficient signal/noise ratio, *i.e.*, I/s>1 with a space group of I222 and a unit cell dimension of *a*=173.27Å, *b*=230.11Å, *c*=249.82Å, and α=β=γ=90°. Table 1 summaries the reflections’ intensities and R-factors by shells.

### 2.3 Model building and refinement of the crystal structure

Applying molecular replacement method, we used the cryo-EM structure of ABCG5/G8 (PDB ID: 7JR7) as the starting model in the Phaser to obtain phase information and a structural solution [1,2]. Phaser's solution at the Walker A, Walker B, and Signature motifs was corrected based on precise registry of the previous ABCG5/G8 crystal structure (PDB ID: 5DO7) before Phenix's model refinement [3,4]. The initial model underwent several refinements which finally resulted in a refined model with Rfree and Rwork of 0.338 and 0.309, respectively. Close inspection of the Fo-Fc map (Fig. 3) illustrated two orthogonal electron densities that clearly include the nature of polycyclic rings and are within the van der Waals’ distance from ABCG5_A540_. We refined the structure in the presence of one cholesterol molecule on each ABCG5/G8 heterodimer by testing a series of sterol orientations using the program COOT [5]. Following our final refinement, Rfree and Rwork improved to 0.302 and 0.244, respectively. Eventually, the quality of the model was validated by MolProbity as implemented in Phenix [3,6]. We have also tried to place cholesteryl hemisuccinate (CHS used during purification) or ergosterol (native sterol from yeast), but either ligand led to severe ligand-protein clash; thus cholesterol is the most suitable ligand in this study.

### 2.4 Molecular docking

ABCG5/G8 models were gathered from the protein data bank while the ABCG5/G8 mutants (ABCG5_A540F_/G8 and ABCG5_Y432F_/G8_N564P_) were generated using UCSF MODELLER. PDB ID: 5DO7 was used as the template file. 400 models were developed and subsequently ranked by discrete optimized protein energy (DOPE). The best model was also visually inspected to determine if any disordered regions were found within the transmembrane domain (TMD).

Molecular Docking was done using UCSF Dock6.9 [7] accessed through SBGrid [8]. Protein files were downloaded from the protein data bank or in the case of mutants, were generated through MODELLLER [9]. All protein files had .pdb extension. The ligand files were downloaded through PubChem as SDF files. Chimera was used to visualize all steps of docking [10]. The nucleotide binding domain (NBD) of the protein was removed to minimize the overall size prior to docking, a requirement since dock6 has a size limit. All three-dimensional .pdb and .sdf model files (protein and ligand) were converted to .mol2 during the preparatory steps of docking as charges and hydrogens were added to each. A surface model of ABCG5/G8 with extension .dms was also developed to use for the generation of spheres (using *sphgen* accessory software) denoting the ligand binding site. The spheres were specified to be generated within a 10 Å radius from the center of each dimer interface, where the ligands were located. A box was built in relation to the specified spheres from the previous step. This box was visually inspected to make sure it maximized coverage of the protein surface and the denoted binding site. This box would also allow the denoting of the energy grid which was built through the *grid* accessory software. Two grid files were generated, file extension .nrg allowing for scoring of ligand conformations, while .bmp determined if the atoms from receptor and ligand are overlapping. Allowed amount of Van der Waal overlap was set at 0.75, providing stringent restrictions on overlap. Grid spacing was set at 0.4. The output files from *grid* were fed into Dock6’s minimization feature, where the ligands were shifted in three dimensions in a rigid manor, *i.e.,* no rotation of bonds. This movement of the ligand minimized the ligand energy by placing it in a more energetically favorable position within the placement site and provided the reference position (input file) for which flex docking procedure started from. Flexible docking provided Dock6 with the freedom to rotate any of the ligand's rotatable bonds to better fit potential binding sites. Maximum orientations were tested at 1000, 5000 and 10000, yielding no differences, indicating that the number of max orientations exceeded the amount needed to saturate new poses. Once flex docking was done, three output files were created: scored, orients and conformers. The scored file was opened through chimera's viewdock feature. The poses were ranked by Van der Waal energy (vdw_energy) values *i.e.,* lower is better. This scoring method was chosen after redocking controls on ABCG2 were conducted [11]. Here, we conducted molecular docking of cholesterol and two plant sterols (stigmasterol and sitosterol) on wild type ABCG5/G8 and mutants ABCG5_A540F_ as well as ABCG5_Y432F_/G8_N564P_ [1]. In Fig. 4, both plant sterols appear to have similar binding conformations, having two partitioned clusters. The poses, parameters and docking data for all three sterols are seen in Tables 3-5. Additionally, both plant sterols are lacking the peak horizontal conformation taken by cholesterol which was resolved in the crystal structure as well as shown in the docking simulation [11]. In the A540F mutant, both plant sterols remain clustered similarly at the bottom of the protein, with the polar heads nearing the intracellular interface. On the other hand, cholesterol displays the similar clustering at the lower membrane region however, maintained a horizontal conformation, this time pointing the other way (Fig. 4, Tables 3-5). The wild-type docking extends from the top of the transmembrane domain (TMD), while in the A540F mutant, sitosterol clusters solely at the bottom with the hydroxyl group facing the intracellular interface of the membrane.

### 2.5 Conservation analysis of protein sequences

Conservational analysis started with protein FASTA sequences gathered from Uniprot for ABCG1, ABCG2, ABCG4. ABCG5 and ABCG8 (P45844, Q9UNQ0, Q9H172, Q9H222, Q9H221). These were then pasted individually into NCBI's basic local alignment search tool for proteins (BLASTp), with the search parameters set at 300 species. Although the whole sequences were pasted in BLAST, only the Phenylalanine Highway (PH) was monitored for conservation. The PH was found to be minimum 99% conserved. The remaining percent was lost at times due to different alignments causing a break in sequences which was noticed after manual inspection. In Fig. 5, we see the conservation of ABCG protein's PH in five different mammalian species.

## Ethics Statements

This work did not involve the use of human subjects. The manuscript adheres to Ethics in publishing standard.

## CRediT Author Statement

**Danny Farhat:** Writing – review & editing, Molecular docking; **Fatemeh Rezaei:** Writing – review & editing, x-ray data analysis; **Jyh-Yeuan Lee:** Acquiring research funding, Writing – review & editing, Oversaw project, Crystallization, and collection of x-ray diffraction data; **Danny Farhat** and **Fatemeh Rezaei:** contributed equally.

## Declaration of Competing Interest

The authors declare that they have no known competing financial interests or personal relationships that could have appeared to influence the work reported in this paper.

## Data Availability

dataset number 973 (source of 8CUB structure) (Original data) (SBGrid Data Bank). Crystal Structure of ABCG5/G8 in Complex with Cholesterol (Original data) (RCSB Protein Data Bank). dataset number 971 (source of 8CUB structure) (Original data) (SBGrid Data Bank). dataset number 972 (source of 8CUB structure) (Original data) (SBGrid Data Bank).
